# Observations on Pemphigus

**Published:** 1788

**Authors:** Stephen Dickson

**Affiliations:** Fellow of the College of Physicians, and one of the King's Professors of Physic in the City of Dublin, M. R. I. A. &c.


V. *Observations on Pemphigus. By* Stephen Dickson, *M. D. Fellow of the College of Physicians, and one of the King's Professors of Physic in the City of Dublin, M. R. I. A.* &c. — From *The Transactions of the Royal Irish Academy*, 1787. 4to. Dublin.

Vera experientia nascitur e compluribus observationibus, magna diligentia, attentione & cura notatis, quæ integram morbi historiam, cum omnibus ad rem pertinentibus circumstantiis complectuntur.

HOFFMAN.

PEMPHIGUS is a disease of very rare occurrence, and many physicians, in extensive practice, have never met with an instance of it. However, six have fallen within my observation — three in Scotland, one in England, and two in this kingdom. I mention this circumstance as an apology for writing on this subject: had the same opportunities occurred to men of more enlarged experience, I should have been silent. I am also aware that uncommon cases are not the best subjects for medical inquiry; but they often serve to reflect light on those which are more usual; and besides, whatever affects human nature muſt naturally conciliate our attention.

Our beſt noſologiſt, Dr. Cullen, (to whom, by the by, no inſtance of this diſeaſe ever occurred) has claſſed pemphigus in the order of Exanthemata. This claſſification will certainly appear ſufficiently proper to thoſe who grant this Noſologiſt the latitude he allows himſelf in the arrangement of his genera. When the plague and petechial fever are allowed to be claſſed under different heads, and the thruſh and ſcarlet fever under the ſame head, we need not contend about the place of pemphigus, even though we ſhould find it not to be contagious, ſometimes commencing and continuing without fever, and affecting perſons more than once in the courſe of their lives. Dr. Cullen deſcribes this diſorder as follows: " A contagious fever, " veſicles about the ſize of an almond* appear-

" ing

* Dr. Cullen's words are, " *avellanæ magnitudine*," *i. e.* " of the ſize of a filbert." This was the ſize of ſome of the largeſt veſicles in a well-marked inſtance of pemphigus that occurred lately to the Editor of this Journal at the Weſtminſter General Diſpenſary. In this patient freſh puſtules continued to appear, from time to time, for the ſpace of ſix weeks; but the eruption was confined to the external ſurface of

" ing on the firſt, ſecond, or third day of the " diſeaſe, remaining for many days, and at " length pouring out a thin ichor."—I propoſe to amend his deſcription in the following manner: *A fever, accompanied with the ſucceſſive eruption from different parts of the body, internal as well as external, of veſicles about the ſize of an almond, which become turgid, with a faintly yellowiſh ſerum, and in three or four days ſubſide.* I ſhall only obſerve at preſent, that I am by no means convinced of this diſorder being contagious; that new veſicles ariſe, not only on the firſt, ſecond, or third, but on every day of the diſeaſe; that I have never known them remain for many days; that the fluid they contain does not appear in general to be an ichor or ſanies, but a bland, inodorous, inſipid ſerum; and that inſtead of being poured out, it is moſt commonly abſorbed into the ſyſtem.

No traces of this diſeaſe are diſcoverable in of the body. A more particular account of this caſe, accompanied with an engraving repreſenting the appearance of the veſicles, may be expected ſoon from an ingenious Student of Phyſic (Mr. T. Chriſtie) who means to make this diſeaſe the ſubject of an inaugural diſſertation. — EDITOR.

the the writings either of the Greeks, Romans, or Arabians.

Bontius, in his account of the medicine of the Egyptians, mentions the caſe of his friend Cavallerius, who was ſeized with the epidemic dyſentery that prevailed during the ſiege in Java, by Tommagon Bauraxa, in 1628. His diſorder was accompanied with the eruption of cuticular veſicles, which were filled with a greeniſh pus, that eroded the ſkin underneath, even to the fleſh. The patient died. It is evident that little can be concluded from this brief account.

Carolus Piſo, in his 149th obſervation, accurately depicts the genuine pemphigus, as it appeared in the caſe of Egmont de Rinach, about an hundred and fifty years ago, at Nantz. He terms it hydatids, and ſays it occurred to him frequently: but I have reaſon to ſuſpect that he confounds under the ſame name the chicken pox, a ſlight diſorder, in which the ſkin is affected, not with ſpreading veſicles, but with ſmall puſtules. He ſeems alſo to confound with pemphigus ſome other erythematous affections; for he ſays that theſe watery puſtules frequently precede the eruption of the itch; that they

ſometimes

ſometimes occur without fever, ſometimes accompany continued fever, and ſometimes appear in the beginning of intermittents. The truth is, that Piſo, though an induſtrious obſerver and a candid man, was by no means an acute noſologiſt. His account, however, of the caſe of Egmont de Rinach deſerves attention, not only as being the firſt accurate and authentic deſcription of this diſeaſe on record, but as pointing out a diverſity in the habit of body then accompanying this diſeaſe from what has been ſince met with; for though Piſo declares that the veſicles in this caſe ſupervened on a putrid ſynochus, yet he ſays that he let blood in the beginning with great advantage, and earneſtly recommends the ſame practice in ſimilar caſes. In every inſtance, however, that I have ſeen of this diſorder, ſuch a practice would have been plainly improper, if not pernicious.

The next author who mentions pemphigus is Morton. Speaking of the diſeaſes which prevailed in London between 1682 and 1692, he mentions, among other fevers of a malignant type, ſome in which watery veſicles were ſcattered over the head and cheſt. Theſe fevers, however, he ſays, were merely ſporadic, and not not propagated by contagion, as in the peſtilential conſtitution.

For the next authentic * account of pemphigus we are indebted to the obſervations of Sauvages. He firſt obſerved it in the hoſpital at Montpellier, in 1725, in a ſoldier who fell a victim to it. Afterwards he ſaw five other caſes, chiefly of beggars, or other poor people, in all of which acute febrile ſymptoms were preſent. Twice, however, he ſaw it unattended with fever.

Laſtly, Dr. Stewart, of Aberdeen, (in a letter to Dr. Duncan, which is inſerted in the Medical Commentaries for 1778) mentions a caſe of pemphigus which occurred to him in the hoſpital in that town. A ſoldier had been ordered to march ſoon after he had been ſeized with the meaſles; the eruption was driven in by the cold, and in ten days afterwards the pemphigus appeared. The veſicles (the largeſt of which were ſnipped) poured out, at firſt, a ſemipellucid ſerum, but in the courſe of the diſeaſe diſcharged a bloody ichor. In this caſe the tendency to putrefaction was very ſtrong; but the patient recovered by the liberal admini-

* See Culleni Noſol. Tom. II. G. xxxiv.

ſtration of bark and wine. From this caſe I think we are juſtified in inferring that the nature of the fluid contained in the veſicles (however accurately it may be aſcertained to be a pure ſerum in the beginning) may be ſo altered in the courſe of the diſeaſe, by its own fermentation, or by admixture with other fluids of the body, from their veſſels being broken down, that it may at length ceaſe to be a diagnoſtic ſymptom of this diſorder.

But no author who has written on the ſubject of pemphigus has mentioned an extraordinary peculiarity of this diſorder, which I have obſerved in two inſtances; namely, that the veſicles have taken poſſeſſion of the internal parts of the body, and proceeded in ſucceſſion (ſome riſing while others decayed) from the mouth, downwards, through the whole ſurface of the alimentary canal.

The firſt caſe in which I had the opportunity of obſerving this ſingular and diſtreſſing ſymptom, was that of a woman under the care of Dr. Gregory, at the Infirmary of Edinburgh, in 1783. This woman's menſes had been obſtructed for two years and a half. During that period ſhe had been thrice before attacked with the ſame diſorder, which had each time ſuper-

 vened vened upon a vomiting of blood. Her ſkin was generally cool, and her pulſe (though weak) never much increaſed in frequency. Peruvian bark and wine were adminiſtered to her liberally: by theſe and other occaſional remedies ſhe recovered.

The other caſe, in which veſicles appeared to have been formed internally, occurred to me lately in this town. I ſhall relate the particulars of it, as I think it worthy of obſervation.

——— ———, aged twenty-three, of a delicate form and ſanguine temperament, the wife of a man in tolerably good circumſtances, and who had been about a fortnight ill of a low fever, was ſeized (after having ſuffered much fatigue in attending her huſband) with pains in her back, head-ach, and tendency to vomit.—As I was attending her huſband I ſaw the firſt approaches of her diſorder, and, on the evening of the day ſhe was firſt attacked, directed her to take an emetic, and to bathe her feet in warm water.

The next morning her ſkin was very hot; pulſe frequent; head-ach not better; ſhe had not ſlept, and complained of a ſore throat: on inſpection the uvula and tonſils appeared inflamed, and ſome mucus was collected in the back back of the fauces: ſhe had had no ſtool for two days. I ordered a clyſter immediately; afterwards a gentle purgative; tincture of roſes for a gargle. In the evening all the ſymptoms were milder. The phyſic had operated twice. I ordered the pediluvium to be repeated.

Third day. She complained of a ſmarting, itching, and (as ſhe expreſſed herſelf) tingling pain in her tongue and through the whole inſide of her mouth. Her tongue was of a bright red colour, and dry, but clean. She was thirſty; but complained that her drink was unpalatable, though acidulated with lemon juice. She had no moiſture on her ſkin: had gone to ſtool once: ſlept tolerably well the night before: the febrile ſymptoms were mitigatad, but the cynanche unabated. I ordered nothing but the ſaline julep.

Fourth day. There appeared on her tongue a pellucid veſicle of about an inch long, and near half an inch broad, turgid with a faintly yellowiſh ſerous fluid. A ſmaller one of the ſame kind appeared on the inſide of the left cheek. The ſenſation which they occaſioned ſhe deſcribed as being ſimilar to that which ſhe had experienced before their eruption, but greater in degree, and ſomewhat as if they were full of ſcalding water. This day her ſkin was cooler; but her pulſe very weak, irregular, and about ninety in a minute. She had had two looſe ſtools. I preſcribed half a drachm of the red Peruvian bark, very finely powdered, to be taken every two hours in a goblet of wine and water. Imperial for common drink. The tincture of roſes to be changed for an emollient gargle.

Fifth day. Three veſicles ſimilar to the former appeared on her cheſt and right arm. Other ſymptoms nearly as before. Pulſe not ſo feeble. Medicines were continued.

Sixth day. Her ſtomach rejected the bark. Two new veſicles appeared on her neck and cheek. Her breath was fœtid. She had had ſome low delirium in the night. Pulſe eighty-eight, and very weak. No ſenſe of taſte. I preſcribed a decoction of bark, one ounce, in which ſhould be diſſolved half a drachm of vegetable alkali, to be taken every two hours; and immediately after each doſe half an ounce of the ſame decoction mixed with ſix drachms of lemon juice. Cyder or porter for common drink.

Seventh day. There was little change. The medicines were continued.

Eighth

Eighth day. The veficles on the infide of the mouth, and on the tongue, difappeared, and the cuticle which had been elevated was fhrivelled, and of a brownifh colour. Deglutition was difficult, and, as fhe faid, painful through the whole infide of her throat. Pulfe eighty, and rather ftronger. Bowels regular. Medicines were continued.

Ninth day. The cuticle on the parts formerly occupied by veficles on the infide of the cheek, and on the tongue, had cracked, and was peeling off: the parts underneath appeared raw and fore. Deglutition had now become fo painful, that fhe refufed medicine, food, and even drink. She could not bear the flighteft preffure on the neck. A new veficle appeared under her right ear. Some purulent matter appeared on the back of the pharynx, the origin of which, however, was not difcernible. Pulfe eighty-fix, and of nearly the fame ftrength. I prefcribed a clyfter of warm water: after its operation, another of new milk and decoction of bark, equal parts; the fame to be repeated four hours afterwards. At night an anodyne clyfter, with fifty drops of thebaic tincture. White liniment for the fores.

Tenth day. The veſicles on the cheſt and right arm had diſappeared. The ſores of the tongue and cheek were of a darker colour, and ſeemed to be healing. Some new veſicles appeared on the abdomen. Pulſe not ſo weak. She reſted well the former part of the preceding night; but was diſturbed by an accident, and afterwards was much inclined to rove in her diſcourſe till morning. Medicines were continued.

Eleventh day. The ſymptoms were nearly the ſame as the day before. The veſicles on the neck and cheek had diſappeared, and the cuticle in thoſe parts was ſhrivelled and cracked. The epigaſtric region was extremely ſore, and this ſoreneſs much increaſed by preſſure. The laſt clyſter of decoction of bark and milk adminiſtered the day before was not retained. I ordered ſalep to be ſubſtituted for milk; other medicines to be continued.

Twelfth day. She could ſwallow, though ſtill not without pain. I directed the medicines which had been preſcribed the fourth day to be repeated; the others to be omitted.

Thirteenth day. She vomited ſome blood along with the firſt doſe of the bark. Pulſe eighty, and ſtronger. The veſicles under the ear and on the abdomen had diſappeared. Several ſmall veſicles (not above the ſize of a pea) aroſe on the hypogaſtric region of the abdomen, one on the labia pudendorum, and two on the left thigh. As ſhe had taken ſome bark, which remained on her ſtomach, I directed this medicine to be continued, and an anodyne draught to be adminiſtered at night.

Fourteenth day. She had two looſe ſtools, much intermixed with blood, and complained of great ſoreneſs of her belly, increaſed by preſſure. I preſcribed a little caſtor oil: other medicines as before, except the draught.

Fifteenth day. She had had two ſtools ſomewhat bloody the night before, and one almoſt natural in the morning. Pulſe ſeventy-ſeven, and of pretty good ſtrength. Skin quite cool; ſpirits better; and ſome little appetite. Menſes had appeared in the morning. I directed the medicines to be continued as before.

From this time ſhe recovered apace, and in about a week had no complaint but weakneſs. Exerciſe, however, and the country air, ſoon completely re-eſtabliſhed her health.

After this full ſtatement of a caſe very diſtinctly marked, it would be ſuperfluous to add any thing by way of comment. I have only to to obſerve, that whether this diſorder be conta-gious or not is a queſtion which may poſſibly ſtill admit of ſome doubt; though, from what I have ſeen, or been able to collect, I am inclined to think that it is not. Almoſt all the inſtances of this diſorder, which are preciſe and well atteſted, I have enumerated; and they are all ſolitary examples, no two of them having happened at the ſame time or place. I ſuſpect, therefore, that ſome other diſorders have been oftentimes miſtaken for pemphigus; and that from thence, or from ſome pre-conceived theory, the notion has ariſen. When I was aſſiſtant to Dr. Home, in the clinical ward of the Infirmary at Edin-burgh, a patient was ſent to us by Dr. Gregory, whoſe caſe he "ſuppoſed * to be a beginning "pemphigus," and which he ſaid "was plain-"ly contagious." In a note which he ſent with this woman, he ſays, "I ſaw a boy five months "ago, in the ſame cloſe, very ill of the ſame "diſeaſe; and I am told by the people that

* Though the diſorder of this patient appeared eventually to be of a different nature, yet it muſt be remembered that the approaches of moſt diſeaſes are ambiguous, and that this ſuppoſition by no means tends to impeach the judgement of a gentleman who is equally diſtinguiſhed for his ſkill and ve-racity.

"ſeveral

" ſeveral others, chiefly children, have had the " ſame diſeaſe ſince in the ſame cloſe." This appeared extremely forcible, and accordingly had its due weight with the ſtudents: but in a day or two it appeared very evidently that the diſeaſe of the woman whom Dr. Gregory had ſent us was merely topical. She had no fever. The veſicles (which were ſituated under the eye and upon the eyelid) were of a pale red colour; ſome puſtules filled with yellow matter appeared upon the brow at the ſame time; and both of theſe vaniſhed almoſt immediately after ſhe came into the Infirmary; ſo that ſhe left it in three or four days perfectly well, having taken no medicine but the ſaline julep. This woman denied to us that ſhe had ever ſeen any one affected with veſicles; and, upon inquiring more particularly among different people in the ſame cloſe, I found that they were in general very unqualified to give a diſtinct account of the epidemic diſeaſe (whatever it was) with which the children had been affected: they ſeemed, however, to think it neither novel nor alarming; and, by their deſcription, I ſhould rather take it to have been the chicken pox, or ſome ſuch ſlight complaint, than the pemphigus. I can have no doubt that the boy Dr. Gregory mentioned he had ſeen was really affected with pemphigus ; but I think that the vague teſtimony of the ignorant, indiſcriminating people of the cloſe is to be allowed no weight in deciding this nice queſtion.

The nature of this diſorder, as to its mildneſs or malignity, appears to vary conſiderably. In ſome inſtances it is extremely mild, as in three of the caſes I have ſeen ; one of them in this town with Dr. Fleury. In other inſtances life is in the greateſt danger ; thus in ſeveral of the caſes I have enumerated ſtrong ſymptoms of putreſcency were manifeſted.

With reſpect to the method of cure of this diſorder, the general ſymptoms of weakneſs and tendency to putrefaction obviouſly point out the proper treatment. When the veſicles ſeize on the internal parts, irritation muſt be guarded againſt by opiates, demulcents, and gentle laxatives ; nouriſhment muſt be ſupplied ; and the grand remedies, bark and wine, (eſpecially the latter) muſt be ſeduloufly adminiſtered.

